# Cytokine Elevation in Severe COVID-19 From Longitudinal Proteomics Analysis: Comparison With Sepsis

**DOI:** 10.3389/fimmu.2021.798338

**Published:** 2022-01-12

**Authors:** Takeshi Ebihara, Hisatake Matsumoto, Tsunehiro Matsubara, Yuki Togami, Shunichiro Nakao, Hiroshi Matsuura, Takashi Kojima, Fuminori Sugihara, Daisuke Okuzaki, Haruhiko Hirata, Hitoshi Yamamura, Hiroshi Ogura

**Affiliations:** ^1^ Department of Traumatology and Acute Critical Medicine, Osaka University Graduate School of Medicine, Suita, Japan; ^2^ Osaka Prefectural Nakakawachi Emergency and Critical Care Center, Higashiosaka, Japan; ^3^ Laboratory for Clinical Investigation, Osaka University Hospital, Suita, Japan; ^4^ Core Instrumentation Facility, Immunology Frontier Research Center and Research Institute for Microbial Diseases, Osaka University, Osaka, Japan; ^5^ Genome Information Research Center, Research Institute for Microbial Diseases, Osaka University, Osaka, Japan; ^6^ Department of Respiratory Medicine and Clinical Immunology, Osaka University Graduate School of Medicine, Suita, Japan

**Keywords:** biomarkers, COVID-19, cytokines, GDF-15, IL-6, mechanical ventilation

## Abstract

**Introduction:**

Coronavirus disease 2019 (COVID-19) is a new viral disease. Uncontrolled inflammation called “cytokine storm” is reported to contribute to disease pathogenesis as well as sepsis. We aimed to identify cytokines related to the pathogenesis of COVID-19 through a proteomics analysis of 1463 plasma proteins, validate these cytokines, and compare them with sepsis.

**Materials and Methods:**

In a derivation cohort of 306 patients with COVID-19, 1463 unique plasma proteins were measured on days 1, 4, and 8. Cytokines associated with disease severity and prognosis were derived. In a validation cohort of 62 COVID-19 patients and 38 sepsis patients treated in the intensive care unit [ICU], these derived cytokines were measured on days 1 (day of ICU admission), 2-3, and 6-8 (maximum: 3 time points/patient). Derived cytokines were compared with healthy controls and between COVID-19 and sepsis patients, and the associations with prognosis were evaluated. The time to wean off mechanical ventilation (MV) was evaluated only for COVID-19.

**Results:**

IL-6, amphiregulin, and growth differentiation factor (GDF)-15 were associated with disease severity and prognosis in the derivation cohort. In the validation cohort, IL-6 and GDF-15 were elevated in COVID-19 and sepsis on day 1, and the levels of these cytokines were higher in sepsis than in COVID-19. IL-6 and GDF-15 were associated with prognosis in sepsis. Cox proportional hazards model with time as a dependent covariate showed a significant relationship between plasma GDF-15 level and time to wean off MV (hazard ratio, 0.549 [95% confidence level, 0.382–0.789]). The GDF-15 level at ICU admission predicted late recovery.

**Conclusion:**

GDF-15 and IL-6 derived from proteomics analysis were related with disease severity of COVID-19. Their values were higher in sepsis than in COVID-19 and were associated with prognosis in sepsis. In COVID-19 patients treated in the ICU, GDF-15 was associated with the time to wean off MV and better predicted late recovery.

## Introduction

Coronavirus disease 2019 (COVID-19), a new viral disease caused by severe acute respiratory syndrome coronavirus-2 (SARS-CoV-2), was first reported in China (1) in December 2019 and has rapidly spread globally, infecting over 262,000,000 people and causing over 5,200,000 deaths as of 1 December 2021 (2). As with sepsis, inappropriate host immune response caused by SARS-CoV-2 can lead to excessive inflammation (3–6) called “cytokine storm” (7). Vascular endothelial damage and thrombotic complications leading to acute respiratory distress syndrome (ARDS) and multiple organ dysfunction syndrome have been reported (8, 9). Circulating cytokines were reported to be important as therapeutic and prognostic biomarkers in COVID-19 (10, 11).

Patients with COVID-19 frequently require prolonged mechanical ventilation (MV) due to refractory pneumonia and ARDS. Nearly 30% of the patients of COVID-19 with MV required tracheostomy due to prolonged MV (12). An observational study evaluating 1890 patients with COVID-19 with tracheostomy in Spain revealed that the median day of tracheostomy was 12 days after intubation and that 24% of these patients remained on MV support after one month (13). Prolonged MV management can lead to long-term hospital stays and vast use of intensive care unit (ICU) resources, thus taking beds away from patients with other diseases that usually require ICU management. In fact, increased mortality from other diseases has been reported during the COVID-19 pandemic (14, 15).

Recently, technological advancements in proteomics have allowed comprehensive analyses of circulating proteins, including cytokines (16, 17). We aimed to identify cytokines related to the pathogenesis of COVID-19 through a proteomics analysis of over 1400 plasma proteins and compare these cytokines with sepsis.

## Materials And Methods

### Derivation Approach Using Public Proteomics

We used publicly available data provided by the Massachusetts General Hospital (MGH) Emergency Department COVID-19 Cohort (18) (Filbin, Goldberg, Hacohen) with Olink Proteomics (https://www.olink.com/mgh-covid-study/) and call this data the MGH cohort. Patients were classified by acuity levels A1-A5 on days 1, 4, 8, and 29 (based on the World Health Organization [WHO] ordinal outcomes scale (19): A1, died; A2, intubated, survived; A3, hospitalized on oxygen; A4, hospitalized without oxygen; A5, discharged). Acuity_max_ was defined as the maximum Acuity score from day 1 through day 29. In this study, we defined “critical” patients as those with Acuity_max_ = A1 or A2. In total, 1472 plasma proteins, including 1463 unique proteins (Olink^®^ Explore 1536), were evaluated with 4 panels, including inflammation, oncology, cardiometabolic, and neurology proteins (20). The levels of protein were expressed as normalized protein expression value (NPX) in log2 scale. In this study, cytokines were defined as “interleukins, interferons, chemokine, colony-stimulation factors and growth factors” (21).

### Validation Approach

As the validation cohort, a prospective observational multicenter study was conducted at the Department of Traumatology and Acute Critical Care Medicine, Osaka University Graduate School of Medicine and Osaka Prefectural Nakakawachi Emergency and Critical Care Center from August 2020 to December 2020. All patients were diagnosed as having RT-PCR-confirmed SARS CoV-2 and pneumonia based on computed tomography (Osaka cohort). To compare with the sepsis pathogenesis, patients with sepsis in a retrospective cohort managed at the Department of Traumatology and Acute Critical Care Medicine, Osaka University Graduate School of Medicine between February 2014 to July 2015 were used. All sepsis patients were >18 years old and fulfilled the Sepsis-3 criteria. The healthy control population comprised outpatients recruited *via* public poster advertisements.

Demographic variables [age, sex, body mass index (BMI)], comorbid conditions (hypertension, diabetes, hyperlipidemia), and clinical variables [laboratory data, Acute Physiology and Chronic Health Evaluation (APACHE) II score, Sequential Organ Failure Assessment (SOFA) score, the day of weaning off MV, and mortality] were extracted from electronic medical records by the investigators.

Patient blood samples were collected on days 1 (day of ICU admission), 2-3, and 6-8 (maximum of 3 time points/patient) and once from the healthy controls. Plasma samples were stored at -30°C until use.

ELISA assays (R&D Systems, Minneapolis, MN, USA) were performed to measure the plasma levels of interleukin (IL)-6, amphiregulin, and growth differentiation factor 15 (GDF-15). Frozen plasma samples were thawed, and subsequent measurement processes were conducted according to the manufacturer’s protocol. Absorbance was analyzed using a microplate reader (SH-9000Lab; Corona Electric Co., Ltd., Japan). Minimum detectable levels were <9.4 pg/mL for IL-6, 15.6 pg/mL for amphiregulin, and 7.8 pg/mL for GDF-15.

The blood samples from the patients were systematically measured by the central laboratory at each hospital to obtain the laboratory data.

This study was conducted according to the principles of the Declaration of Helsinki and was approved by the institutional review board of Osaka University Hospital [Approval numbers: 12007, 16109 and 885 (Osaka University Critical Care Consortium Novel Omix Project; Occonomix Project)]. Informed consent was obtained from the patients or their relatives and the healthy volunteers for the collection of all blood samples.

### Definition of Early or Late Recovery

The median time to wean off MV was 12 days after intubation in the Osaka cohort (**Table 2**), and the median day of tracheostomy after intubation was reported to be day 12 in a large Spanish observational study (13). Accordingly, MV for ≤12 days was defined as early recovery, and MV >12 days or hospital death was defined as late recovery in this study.

### Statistical Analysis

Values are reported as n (%) and median (quartiles 1-3).

In the MGH cohort, the values of age and BMI and comorbidities were compared between the critical and non-critical patients by chi-square test. The NPXs for each protein were compared between critical patients (Acuity_max_ = A1, A2) and non-critical patients (Acuity_max_ = A3, A4, A5) on days 1, 4, and 8. The results were filtered using the Benjamin-Hochberg procedure for false discovery rate (FDR) correction. Data are shown with a volcano plot. The X-axis shows differences in the NPX values, and the Y-axis shows the -log10 (FDR). A statistically significant difference was defined as FDR <0.01 and differences in the NPX values >1.0. Cytokines reaching significance from day 1 to day 8 were analyzed using receiver operating characteristic (ROC) curves to determine whether the day 1 NPX was useful as a prognostic biomarker (Acuity_max_ = A1) or marker of disease severity (Acuity_max_ = A1, A2). Area under the curve (AUC), accuracy, sensitivity, and specificity were also measured. Values with AUC >0.7 for both prognosis and disease severity were included in the validation cohort.

In the Osaka cohort, the values of age, sex, and BMI and comorbidities were compared between three groups by Kruskal-Wallis test and chi-square test. The clinical and demographic characteristics between COVID-19 and sepsis were compared by Wilcoxon rank-sum test or chi-square test. The plasma IL-6, amphiregulin, and GDF-15 levels were transformed to logarithm values to normalize data distribution before the analyses. Dunnett’s test was used to evaluate differences in each value between the patients and healthy controls. The Wilcoxon rank-sum test was used to evaluate differences between survivors and non-survivors on each day for COVID-19 and sepsis. For COVID-19, further analyses were performed. The patients were divided into two groups in the acute phase (day 1, days 2-3, and days 6-8): early recovery and late recovery. The Wilcoxon rank-sum test was used to evaluate differences between the two groups on each day. A Cox proportional hazards model with time as a dependent covariate was applied to assess the association of IL-6, amphiregulin, and GDF-15 with the time to wean off MV. The hazard ratios are shown as Z-scores to allow comparison of the strength of the association between biomarkers. The event was weaning off MV. A hazard ratio <1 means that an increase of the biomarker is associated with longer time until weaning off MV. To investigate whether the day 1 IL-6, amphiregulin, GDF-15, CRP, neutrophil-to-lymphocyte ratio, and lactate dehydrogenase (LDH) values were useful biomarkers for predicting late recovery, we created ROC curves, and the AUC, accuracy, sensitivity, and specificity were determined.

P values <0.05 were considered to indicate statistical significance. The data were analyzed using R version 4.0.2 (R Foundation for Statistical Computing, Vienna, Austria) and are presented using Graph Pad Prism, version 8.4.3 (GraphPad Software, La Jolla, CA, USA).

## Results

### Overview

**Figure 1:**
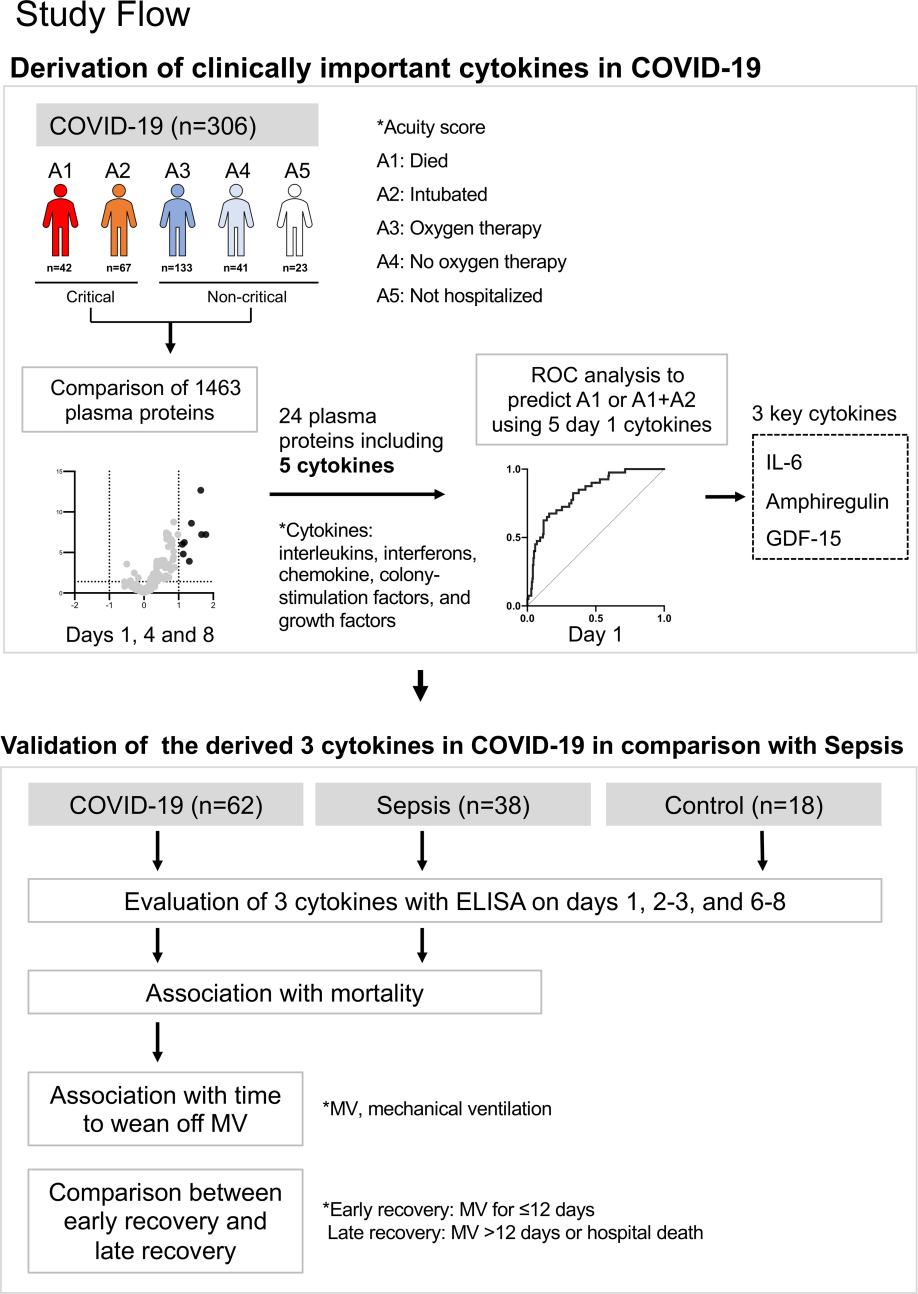
Summary of this study. The first goal was to determine clinically important cytokines in COVID-19, and the second goal was to validate these cytokines in comparison with those of sepsis.

The study approach involved two datasets and a statistical approach (**Figure 1**). The first goal was to determine clinically important cytokines in COVID-19, and the second goal was to validate these cytokines in comparison with those of sepsis.

### Derivation of Clinically Important Cytokines in COVID-19

In the MGH cohort, one of the 306 of patients with COVID-19 was flagged as an outlier and removed from the final dataset, leaving 305 day 1 samples, 215 day 4 samples, and 139 day 8 samples. Overall, 42 patients died within 28 days and 263 survived to 28 days, and 196 patients were critical (Acuity_max_ = A1, A2) and 109 were non-critical (Acuity_max_ = A3, A4, A5). The distribution of patients by age group was statistically different between the critical and non-critical patients. Other characteristics are shown in **Table 1**.

**Table 1:** Clinical and demographic characteristics of COVID-19 patients in the MGH cohort.

	Critical (A1, A2)	Non-Critical (A3, A4, A5)	P-value
	(n=109)	(n=196)	
Age group, n (%)			<0.01
Under 65 years	45 (41.3)	141 (71.9)	
65-79 years	37 (33.9)	28 (14.3)	
80 years or over	27 (24.8)	27 (13.8)	
BMI group, n (%)			0.19
Under 25.0	19 (17.4)	27 (13.8)	
25.0-39.9	73 (67.0)	131 (66.8)	
Over 40.0	13 (11.9)	22 (11.2)	
Unknown	4 (3.7)	16 (8.2)	
Comorbidities, n (%)			
Hypertension	65 (59.6)	81 (41.3)	<0.01
Diabetes	50 (45.9)	60 (30.6)	<0.01
28-day death, n (%)	42 (38.5)	0 (0.0)	

Data are given as number (%). WHO ordinal outcomes scale: A1, died; A2, intubated, survived; A3, hospitalized on oxygen; A4, hospitalized without oxygen; A5, discharged. BMI, body mass index; MGH, Massachusetts General Hospital.

**Figure 2:**
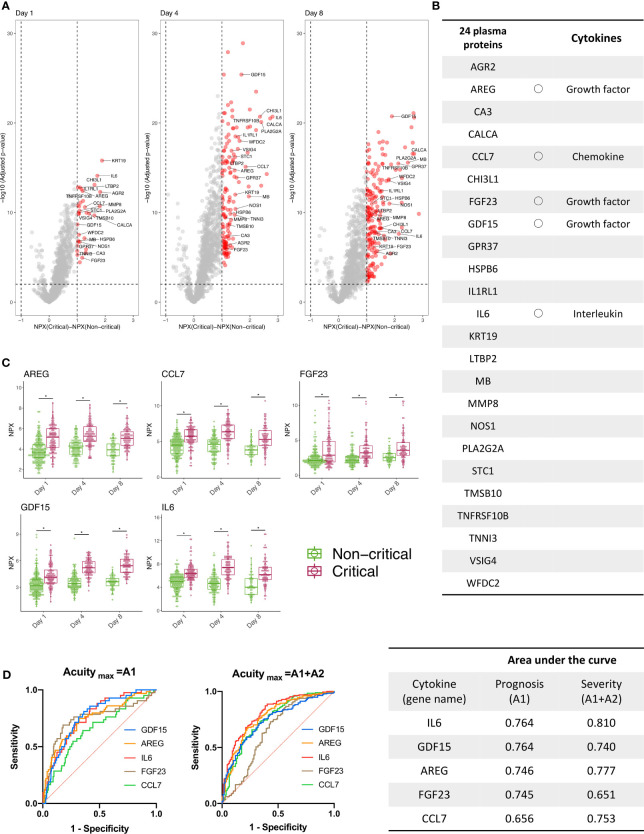
Three cytokines were derived using public proteomics data provided by the MGH COVID-19 cohort. **(A)** The volcano plot shows the proteins increased (red) or decreased (blue) in patients with critical COVID-19 (Acuity_max_ = A1, A2) versus patients with non-critical COVID-19 (Acuity_max_ = A3, A4, A5) at days 1 (day of admission), 4, and 7 in the MGH derivation cohort. The X-axis shows the differences in NPX, and the Y-axis represents -log10 (adjusted P-values). **(B)** Twenty-four proteins were classified as significantly increased and were the proteins that showed differences of NPX >2 and -log10 (adjusted P-values) >2 from day 1 to day 8. Five of the 24 proteins (gene names: AREG, CCL7, FGF23, GDF15, IL6) were classified as cytokines. **(C)** Longitudinal change of the five cytokines. The COVID-19 individuals were further classified into two groups, “Non-critical “and “Critical”, on days 1, 4, and 8. The NPX values are plotted on the Y axes. In all box plots, the boxes show the median and upper and lower quartiles, and the whiskers show 5th to 95th percentiles. The difference between two groups was measured by Wilcoxon rank-sum test (*P < 0.05). **(D)** The NPXs of 5 cytokines on day 1 were used for an ROC curve analysis, and the AUC was calculated to evaluate the severity and prognostic accuracy of each marker. For the following cytokines with gene names IL6, AREG, and GDF15, the AUCs of both prognosis (Acuity_max_ = A1) and disease severity (Acuity_max_ = A1, A2) were >0.7. WHO ordinal outcomes scale: A1, died; A2, intubated, survived; A3, hospitalized on oxygen; A4, hospitalized without oxygen; A5, discharged). MGH, Massachusetts General Hospital; COVID-19, coronavirus disease 2019; NPX, normalized protein expression value; ROC, receiver operating characteristic; AUC, area under the curve; IL, interleukin; GDF, growth differentiation factor; WHO, World Health Organization.

Proteins that showed statistically significant changes in expression are indicated in red in the volcano plots (**Figure 2A**). All proteins that showed statistically significant changes in expression on days 1, 4, and 8 are shown in **Figure 2B**. Five of the 24 proteins (gene names: AREG, CCL7, FGF23, GDF15, IL6) were classified as cytokines (21). AREG, FGF23, and GDF15 are growth factors, CCL7 is a chemokine, and IL6 is an interleukin. The longitudinal changes of these five cytokines divided between critical and non-critical patients are shown in **Figure 2C**. AUCs of the day 1 NPX of these cytokines for disease severity (Acuity_max_ = A1, A2) and prognosis (Acuity_max_ = A1) were evaluated. For three cytokines with gene names IL6, AREG, and GDF15, the AUC was >0.7 for both prognosis and disease severity (**Figure 2D**).

### Validation of IL-6, GDF-15, and Amphiregulin for COVID-19 and Sepsis Patients

In the Osaka cohort, we enrolled 62 patients with COVID-19 (42 men, 20 women), 38 patients with sepsis (29 men, 9 women), and 18 healthy controls (12 men, 6 women). The median age, age group distribution, sex, and BMI were not significantly different between the three groups (**Table 2**). All patients with COVID-19 were treated in the ICU, and 60 patients (96.8%) were treated with MV. Sepsis patients were also treated in the ICU: 81.6% were treated with the MV and 26.3% had pneumonia. The median APACHE II score and SOFA score in the COVID-19 and sepsis patients were 14 and 21 (P <0.01), and 5 and 9 (P <0.01), respectively. Hospital mortality rates in the COVID-19 and sepsis patients were 12.9% and 26.3% (P = 0.09), respectively (**Table 3**). The comorbidities and laboratory data are shown in **Table 2**.

**Table 2:** Characteristics of healthy controls, COVID-19 and sepsis patients in the Osaka cohort.

	Healthy controls	COVID-19 patients	Sepsis patients	P-value
	(n=18)	(n=62)	(n=38)	
Age	73 (63–77)	71 (61–76)	74 (65–81)	0.18
Age group, n (%)				0.58
Under 60 years	3 (16.7)	15 (21.1)	5 (13.2)	
60-69 years	5 (22.2)	13 (18.3)	9 (23.7)	
70-79 years	7 (38.9)	25 (35.1)	13 (34.1)	
80 years or over	3 (16.7)	9 (14.5)	11 (29.0)	
Sex, male n (%)	12 (66.6)	42 (67.7)	29 (76.3)	0.61
BMI	22.5 (20.7-24.8)	24.1 (22.6-26.3)	21.6 (19.0-23.5)	<0.01
Comorbidities, n (%)				
Hypertension	6 (33.3)	33 (53.2)	11 (28.9)	0.04
Diabetes	1 (5.6)	27 (43.5)	15 (39.5)	0.01
Hyperlipidemia	5 (27.8)	19 (30.6)	7 (18.4)	0.40

Data are given as the median (25th-75th percentile) or as number (%). BMI, body mass index.

**Table 3:** Clinical and demographic characteristics of COVID-19 and sepsis patients in the Osaka cohort.

	COVID-19 patients	Sepsis patients	P-value
	(n=62)	(n=38)	
Laboratory data			
White blood cell (/µL)	7,700 (4,700–14,000)	10,700 (6,800–15,400)	0.02
Platelet count (10^4^/µL)	19.8 (15.9-24.0)	12.0 (4.8-26.6)	<0.01
D-dimer (μg/mL)	2.5 (1.3-4.2)	8.7 (3.8-14.9)	<0.01
Creatinine (mg/dL)	0.7 (0.5-0.9)	1.6 (0.9-2.3)	<0.01
Bilirubin (mg/dL)	0.5 (0.4-0.7)	0.7 (0.5-1.3)	<0.01
CRP (mg/dL)	9.5 (5.3-13.3)	16.0 (7.9-21.6)	<0.01
Origin, n (%)			<0.01
Chest	62 (100)	10 (26.3)	
Abdomen	0 (0)	11 (29.0)	
Soft tissue	0 (0)	12 (31.6)	
Urinary	0 (0)	3 (7.8)	
Others	0 (0)	2 (5.3)	
APACHE II score	14 (9–17)	21 (14–30)	<0.01
SOFA score	5 (3–6)	9 (5–13)	<0.01
MV, n (%)	60 (96.8)	31 (81.6)	0.01
Days to weaning off MV	12 (7–55)	9 (3–15)	<0.01
Mortality, n (%)			
28-days	5 (8.1)	9 (23.7)	0.03
Hospital	8 (12.9)	10 (26.3)	0.09

Data are given as the median (25th-75th percentile) or as number (%). APACHE, Acute Physiology and Chronic Health Evaluation; CRP, C-reactive protein; IQR, interquartile range; MV, mechanical ventilation; SOFA, Sequential Organ Failure Assessment.

**Figure 3:**
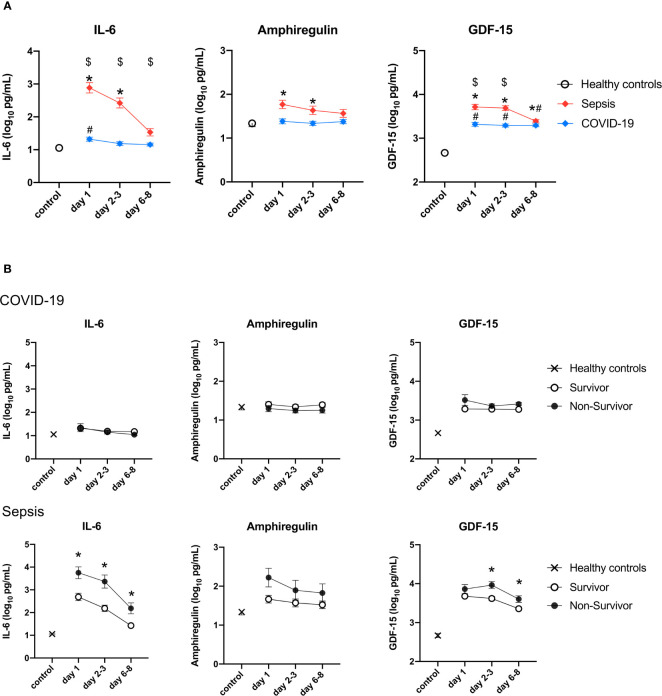
Change in the levels of three cytokines in the validation cohort. The cytokines were transformed to common logarithm values to normalize the data distribution. All data are expressed as the mean ± SE. **(A)** Asterisks indicate a statistically significant difference between control and septic patients (there were significant differences in the three cytokines), # indicates a statistically significant difference between control and with patients COVID-19 on each day (P <0.05), $ indicates a statistically significant difference between patients with sepsis and patients with COVID-19. **(B)** The cytokine levels in survivors and non-survivors on each day in patients with sepsis and patients with COVID-19. Asterisks indicate a statistically significant difference between survivors and non-survivors (P < 0.05) on each day. SE, standard error; COVID-19, coronavirus disease 2019; IL, interleukin; GDF, growth differentiation factor.

In comparison to those of the healthy controls, the plasma GDF-15 levels of the COVID-19 and sepsis patients were significantly higher on days 1, 2-3, and 6-8. The plasma IL-6 levels of the patients with COVID-19 on day 1 and the sepsis patients on days 1 and 2-3, and the plasma amphiregulin levels of the sepsis patients on day 1, were significantly higher than those of the healthy controls (**Figure 3A**). The levels of IL-6 and GDF-15 in sepsis were statistically significantly higher than those in COVID-19 on day 1 to days 6-8, and on day 1 and days 2-3, respectively (**Figure 2A**). There were no differences in the plasma levels of these cytokines between survivors and non-survivors among the patients with COVID-19. However, among the patients with sepsis, plasma IL-6 levels of the non-survivors were significantly higher than those of the survivors from day 1 to days 6-8, as were those of GDF-15 on days 2-3 and 6-8 (**Figure 3B**).

**Figure 4:**
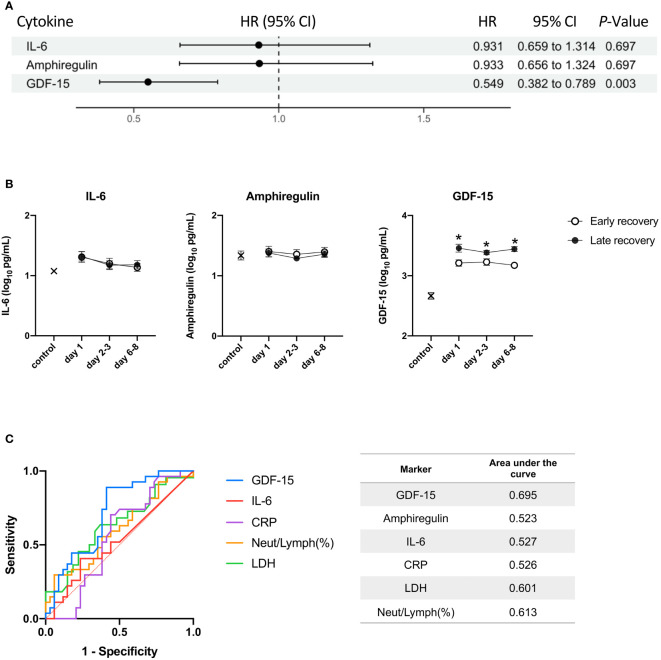
The relationship between the three cytokines and time to wean off MV. The day of weaning off MV was defined as the day of extubation for patients without tracheostomy or coming off the ventilator for patients with tracheostomy. **(A)** A Cox proportional hazards analysis with time as a dependent covariate for weaning off MV. The hazard ratios are provided as Z-scores to allow the strength of association between biomarkers to be compared. Benjamin-Hochberg correction for multiple testing was performed when calculating P values. **(B)** IL-6, amphiregulin, and GDF-15 were transformed to common logarithm values to normalize data distribution. All data are expressed as the mean ± SE. The cytokine levels in patients with early recovery and late recovery on day 1 (n = 35; n = 26, respectively), days 2-3 (n = 34; n = 25, respectively), and days 6-8 (n = 32; n = 26). Asterisks indicate a statistically significant difference between patients with early recovery and patients with late recovery (P < 0.05) on each day. **(C)** The levels of the three cytokines, CRP, LDH, and neutrophil-to-lymphocyte ratio were used for the ROC curve analysis. The AUC was calculated to evaluate the predictive accuracy of each marker on day 1 for predicting late recovery. MV, mechanical ventilation; HR, hazard ratio; CI, confidence interval; IL, interleukin; GDF, growth differentiation factor; SE, standard error; LDH, lactate dehydrogenase; ROC, receiver operating characteristic; AUC, area under the curve.

For COVID-19, Cox proportional hazard analyses with time as a dependent covariate showed that as the P-value for GDF-15 was <0.05, high GDF-15 was associated with longer time until weaning off MV (**Figure 4A**). Plasma GDF-15 levels were significantly higher in late recovery than early recovery from day 1 to days 6-8 (**Figure 4B**). GDF-15 was the most useful marker for predicting late recovery (AUC = 0.695) (**Figure 4C**).

## Discussion

In this study, we derived three cytokines, IL-6, amphiregulin, and GDF-15, that were related to disease severity in COVID-19 and compared these cytokines between COVID-19 and sepsis. All three cytokines were elevated in both COVID-19 and sepsis patients compared with those in the healthy controls. The levels of these cytokines in sepsis were statistically significantly higher than those in COVID-19. IL-6 and GDF-15 were related to prognosis in sepsis. In COVID-19, no cytokines were associated with mortality, and only GDF-15 was associated with late recovery.

In severe cases of COVID-19, excessive inflammation in the lung alveoli leads to severe hypoxia and ARDS and has features of systemic cytokine release syndrome presenting with high fever and abnormal CRP (22). Various pro- and anti-inflammatory cytokines are reported to be increased in the blood of patients with COVID-19-associated ARDS (10, 23), and reductions in type I and III interferons were observed in COVID-19 patients hospitalized for pneumonia (24). Cytokine storm potentially causes COVID-19-associated coagulopathy (25), suggesting that cytokines may be involved in the pathogenesis of COVID-19 and could be a potential therapeutic target (22, 26).

We showed IL-6 to be related to disease severity in mild to severe cases of COVID-19 in the MGH cohort. In contrast, in the Osaka cohort including only severe COVID-19 patients, IL-6 was not associated with prognosis or time to wean off MV. In the present study, IL-6 levels were also measured in sepsis patients and were 10 to 100 times higher than those in the patients with COVID-19 and were associated with prognosis. Although the pathogenesis of COVID-19 is described as producing a cytokine storm (7) involving IL-6, the present study and that of Leisman et al. (27) found that the production of IL-6 in COVID-19 was not dramatically increased compared to that in sepsis.

Recent studies have shown that IL-6 is crucial in the pathogenesis of COVID-19 (10) and IL-6 receptor blockade has been investigated as a potential therapy (28). Importantly, IL-6 has also been reported to play an important role in controlling inflammatory cytokines (29, 30) and to be protective in hyperoxia-induced lung damage (31, 32). In COVID-19, the spike protein of SARS-CoV-2 induces the production of IL-6 by both macrophages and lung epithelial cells (33). The excess or continuous production of IL-6—as seen in lethal sepsis—can be harmful. However, the production of IL-6 could be a normal reaction of the host defense in COVID-19. The effect of IL-6 itself on the pathogenesis of COVID-19 may be complex.

Amphiregulin is a member of the epidermal growth factor (EGF) group expressed in various epithelial cell types. Its roles, including regulation of lung morphogenesis, mammary gland development, and keratinocyte proliferation, seem broad and not fully understood (34). In the context of various inflammatory stimuli, amphiregulin is reported to be induced in various immune cells such as eosinophils, mast cells, basophils, group 2 innate lymphoid cells, and FoxP3-expressing CD4+ regulatory T cells (Tregs) (34). In the present study, amphiregulin was not significantly associated with prognosis or time to wean off MV in the Osaka cohort. Amphiregulin tended to be lower in the late recovery or death groups in the Osaka cohort, which differed from the results in the MGH cohort. Interestingly, Harb et al. reported that amphiregulin levels (as measured by ELISA) of patients with mild COVID-19 were higher than those of healthy individuals and decreased with the severity of the illness (35). Further investigations are needed to clarify the role of amphiregulin in COVID-19.

GDF-15 is a transforming growth factor (TGF)-β molecule superfamily member originally identified in the 1990s (36). In the human basal state, GDF-15 transcripts are expressed in virtually all tissues but show higher prevalence in macrophages, airway epithelial cells, and vascular endothelial cells (37). The roles of GDF-15, including regulation of neutrophil arrest and platelet aggregation and the suppression of hepcidin, a master regulator of iron homeostasis in human hepatocytes, are also broad and not completely understood. The GDF-15 level provides independent prognostic information about cardiovascular disease (38) and lung disease (39). In the present study and in a previous report (40), GDF-15 levels were associated with mortality in sepsis patients. Myhre et al. investigated the association between GDF-15 and outcomes of 123 patients with COVID-19 and reported that higher concentrations are associated with SARS-CoV-2 viremia, hypoxia, and worse outcomes (38). The present study adds information indicating that GDF-15 was associated with late recovery or death and was an important biomarker to predict late recovery or death only in COVID-19 patients treated in the ICU.

The mechanism of high GDF-15 levels in COVID-19 remain unknown. GDF-15-deficient mice were reported to be protected against abdominal sepsis due to increased chemokine CXC ligand 5 (CXCL5)-mediated recruitment of neutrophils into the peritoneum, leading to better local bacterial control (41). Further studies including identification of the site GDF-15 production may clarify whether GDF-15 can be new therapeutic target for critical COVID-19 patients.

This study has several limitations. First, the study population is relatively small. Second, the measuring points are based on the time from admission, and thus, the time from onset was not considered. Third, unmeasured confounders such as treatment details are lacking that might have biased the results. Fourth, the ages between the critical and non-critical patients in the MGH cohort were different but were not considered to affect the derivation of the cytokines. Finally, we compared COVID-19 and sepsis in this study, but the level of disease severity as indicated by measures such as the APACHE II score or SOFA score was not considered.

## Conclusion

We derived cytokines associated with disease severity and prognosis from 1463 plasma proteins—including more than 200 cytokines—in patients with COVID-19. GDF-15 and IL-6 appeared to be related with disease severity of COVID-19, but their levels were higher in sepsis than in COVID-19 and were associated with prognosis in sepsis. In COVID-19 patients treated in the ICU, the GDF-15 level was associated with the time to wean off MV and better predicted late recovery.

## Data Availability Statement

Publicly available datasets were analyzed in this study. This data can be found here: https://www.olink.com/mgh-covid-study/.

## Ethics Statement

The studies involving human participants were reviewed and approved by Osaka University Hospital. The patients/participants provided their written informed consent to participate in this study.

## Author Contributions

TE conceived and designed this study, acquired data, analyzed and wrote the manuscript. HisM helped with designing the study and data interpretation and conducted the literature review. TM, YT, TK, HirM, HH, and HY contributed to data acquisition. FS and DO helped analyze the data. SN helped with designing the study. HO conducted the literature review. All authors have read and understood journal’s policies and believe that neither the manuscript nor the study violates any of these. All authors meet the authorship criteria detailed in the submission guidelines, and all authors agree with the contents of the manuscript. All authors contributed to the article and approved the submitted version.

## Funding

This study was supported by JSPS KAKENHI Grant Number 20K17892 and Japan Agency for Medical Research and Development Grant Number 20fk0108404h0001.

## Conflict of Interest

The authors declare that the research was conducted in the absence of any commercial or financial relationships that could be construed as a potential conflict of interest.

## Publisher’s Note

All claims expressed in this article are solely those of the authors and do not necessarily represent those of their affiliated organizations, or those of the publisher, the editors and the reviewers. Any product that may be evaluated in this article, or claim that may be made by its manufacturer, is not guaranteed or endorsed by the publisher.

## Abbreviations

APACHE, Acute Physiology and Chronic Health Evaluation; ARDS, acute respiratory distress syndrome; AUC, area under the curve; BMI, body mass index; CI, confidence interval; COVID-19, coronavirus disease 2019; GDF, growth differentiation factor; HR, hazard ratio; ICU, intensive care unit; IL, interleukin; LDH, lactate dehydrogenase; MGH, Massachusetts General Hospital; MV, mechanical ventilation; NPX, normalized protein expression value; ROC, receiver operating characteristic; SOFA, Sequential Organ Failure Assessment; WHO, World Health Organization.
